# Native Bacteria Are Effective Biocontrol Agents at a Wide Range of Temperatures of *Neofusicoccum parvum*, Associated with Botryosphaeria Dieback on Grapevine

**DOI:** 10.3390/plants14071043

**Published:** 2025-03-27

**Authors:** Diyanira Castillo-Novales, Paulina Vega-Celedón, Alejandra Larach, Michael Seeger, Ximena Besoain

**Affiliations:** 1 Molecular Microbiology and Environmental Biotechnology Laboratory, Department of Chemistry, Center of Biotechnology Daniel Alkalay Lowitt, Universidad Técnica Federico Santa María, Avenida España 1680, Valparaíso 2390123, Chile; diyaniracastillonovales@gmail.com (D.C.-N.); pvegaceledon@gmail.com (P.V.-C.); alarach.v@gmail.com (A.L.); 2 Escuela de Agronomía, Facultad de Ciencias Agronómicas y de los Alimentos, Pontificia Universidad Católica de Valparaíso, San Francisco s/n La Palma, Quillota 2260000, Chile; 3 Millennium Nucleus Bioproducts, Genomics and Environmental Microbiology (BioGEM), Avenida España 1680, Valparaíso 2390123, Chile

**Keywords:** biocontrol, *Pseudomonas*, *Neofusicoccum parvum*, Botryosphaeria dieback, grapevine trunk disease, *Vitis vinifera* L., Cabernet Sauvignon, Sauvignon Blanc

## Abstract

Botryosphaeria dieback, a significant grapevine trunk disease (GTD) caused by various pathogens, represents a serious threat to viticulture. Biocontrol emerges as a promising sustainable alternative to chemical control, aligning toward environmentally friendly viticultural practices. This study evaluated the in vitro, in vivo, and in situ biocontrol potential of Chilean native bacteria isolated from wild flora and endophytic communities of grapevine against *Neofusicoccum parvum*. In vitro biocontrol assays screened 15 bacterial strains at 10, 22, and 30 °C, identifying four *Pseudomonas* strains with >30% mycelial growth inhibition. In diffusible agar and double plate assays, plant growth-promoting bacteria AMCR2b and GcR15a, which were isolated from native flora, achieved significant inhibition of *N. parvum* growth, with reductions of up to ~50% (diffusible agar) and up to ~46% (double plate). In vivo experiments on grapevine cuttings revealed that strains AMCR2b and GcR15a inhibited mycelial growth (17–90%); younger grapevines (1–5 years) were more susceptible to *N. parvum*. In situ trials using *Vitis vinifera* L. cv. Cabernet Sauvignon and Sauvignon Blanc demonstrated higher fungal susceptibility in Sauvignon Blanc. These results highlight the potential of *Pseudomonas* sp. AMCR2b and GcR15a to be effective biocontrol agents against GTDs at a wide range of temperatures, contributing to sustainable viticulture.

## 1. Introduction

*Vitis vinifera* L. (Linnaeus, [1]) is one of the most important agricultural species worldwide. Cultivated primarily in Mediterranean and temperate climate regions between latitudes 30° and 50°, it spans approximately 7.72 million hectares [2,3,4,5]. This crop underpins the production of wine, table grapes, and highly valued economic and cultural products that sustain the economies of numerous regions worldwide. In agriculture, massive losses have been reported in diverse regions around the world due to phytopathogens and abiotic stresses (salinity, drought, extreme temperatures) that have been intensified by climate change [6,7,8]. The woody agricultural plant *V. vinifera* hosts of various variety of pathogens, especially pathogenic fungi [9,10,11,12,13,14,15,16,17,18,19,20,21,22].

Grapevine trunk diseases (GTDs), caused by fungal pathogens, are currently recognized as one of the most severe challenges for viticulture. These diseases affect vine longevity and significantly limit crop productivity [13,22,23,24,25]. GTDs include various pathologies such as Botryosphaeria dieback (caused by species from the *Botryosphaeriaceae* family such as *Neofusicoccum*, *Diplodia*, *Lasiodiplodia*, and *Dothiorella*), *Eutypa* dieback (caused by the *Eutypa* genus), Esca (caused by *Fomitiporia*, *Stereum*, *Inonotus*, *Phaeomoniella*, and *Phaeoacremonium*), and Black foot (caused by *Ilyonectria, Dactylonectria*, *Campylocarpon*, and *Cylindrocarpon*) [26]. Botryosphaeria dieback, caused by more than 20 species of the *Botryosphaeriaceae* family, has emerged as one of the most significant threats to vineyards worldwide, mainly due to its destructive impact and challenging management [27,28]. In North America, yield losses reach up to 94% in severe cases of Botryosphaeria damage [29]. In Europe, the incidence of these diseases has risen dramatically over recent decades. In France, annual losses due to GTDs are estimated at one billion euros, affecting approximately 12% of vineyards [30]. In Italy, Esca impacts up to 80% of mature vineyards in the southern regions [31], while Spain experienced an increase in GTD incidence from 1.8% to 7% between 2001 and 2006 [32]. In Asia and North America, China and Canada also report alarming increases in GTD incidence, with up to 90% of vineyards affected in British Columbia [4,33,34].

In Chile, where viticulture represents a key economic and cultural activity [5], GTDs have had a significant impact, particularly in the central region, which accounts for most of the country’s wine production. Recent studies have reported an alarming incidence of up to 87% in Cabernet Sauvignon vineyards, with yield losses increasing from 39% to 46% in less than a decade, underscoring growing impact and persistence of these pathogens in viticultural systems [35]. In the valleys of Central Chile, where temperate climatic conditions favor its development and spread [36], *Neofusicoccum parvum* (Pennycook & Samuels [37] and Crous et al. [38]) is one of the most prevalent pathogens in various fruit crop varieties. In Chile, GTDs in wine vines and table grape have been predominantly associated with ascomycete fungi from the *Botryosphaeriaceae* family, including *N. parvum*, *Diplodia seriata*, *Diplodia mutila*, *Neofusicoccum australe*, *Phaeomoniella chlamydospora*, *Diaporthe ambigua*, and *Spencermartinsia viticola* [35,39,40,41,42,43,44,45,46].

Within the *Botryosphaeriaceae* family, *N. parvum* is recognized as one of the most aggressive and prevalent pathogens. It affects the permanent woody structures of grapevines, including the arms, trunks, and cordons, causing internal cankers and necrosis, ultimately leading to shoot and bud dieback—commonly known as “dieback” [11,47]. Its ability to rapidly colonize wood and adapt to diverse environmental conditions threatens vineyard sustainability [36,48,49]. The ability of *N. parvum* to adjust to varying temperatures, particularly within the range of 25 °C to 28 °C [36], establishes it as a highly adequate pathogen in temperate climates such as those in Central Chile. Studies have demonstrated that its mycelial growth is significantly inhibited at extreme temperatures, such as 10 °C and 35 °C [50]. The thermal adaptability of *N. parvum* may reflect an evolutionary adjustment to the cooler temperatures typical of the valleys of Central Chile during spring and summer. Such a trait may contribute to the notable prevalence and virulence of the pathogen in this region [35].

Diverse strategies have been applied to control phytopathogens, including chemical control [26,35,51], the use of rootstocks [8], biological control [52,53], and the application of plant compounds [54]. The rich biodiversity of diverse ecosystems in Chile is a valuable and largely unexplored source of microorganisms useful for a wide range of biotechnological applications [52,55]. Beneficial microorganisms promote growth of agricultural crops and may be biological control agents (BCAs) for managing phytopathogens [5,52,56,57,58,59,60,61,62]. Plant growth-promoting bacteria (PGPB) (e.g., *Pseudomonas*, *Bacillus*, *Paraburkholderia*, and *Halomonas*) and fungi (e.g., mycorrhiza, *Trichoderma*) stimulate plant growth and may control phytopathogens. BCAs have demonstrated effectiveness against various grapevine trunk pathogens, offering a more environmentally friendly treatment [4,26,63,64,65,66,67]. The control of *N. parvum* relies mainly on pruning infected wood and application of fungicides. Banning of fungicides due to environmental and health concerns has reduced available control treatments, increased management costs, and decreased the efficacy of chemical control [26,51,68]. Therefore, biocontrol of *N. parvum* by bacteria and fungi is of increasing interest [4]. *B. subtilis* strains isolated from grapevine wood and the rhizosphere have demonstrated both direct and indirect protection against *N. parvum* through antibiotic production and the induction of plant immunity [69,70]. In vitro assays have shown that *Bacillus velezensis*, *Bacillus* sp., and *Pseudomonas chlororaphis* strains inhibit *N. parvum* through agar-diffusible metabolites and volatile organic compounds [51,71]. However, other bacteria (e.g., *Paenibacillus*) also may contribute to fungal grapevine wood degradation [72].

This study aimed to evaluate the biocontrol potential of 15 native bacteria against *N. parvum* strains by analyzing their efficacy at different temperatures in in vitro assays. Different native bacterial and fungal strains isolated from diverse ecosystems in Chile [35,52,62] were selected for this study. Two bacterial strains were subsequently selected for in vivo tests using grapevine cuttings of different ages and in situ experiments in *V. vinifera* vineyards. This study contributes to the development of sustainable management strategies to mitigate the impact of *N. parvum* on Chilean viticulture, promoting environmentally friendly practices.

## 2. Results

### 2.1. In Vitro Dual Antagonism Assays of Bacteria Against Grapevine Trunk Pathogens

Fifteen native bacterial strains were evaluated after seven days, for their ability to inhibit the mycelial growth of *N. parvum* strains (PUCV 1547, PUCV 1557, and PUCV 1560). Four bacterial strains—GcR15a, AMCR2b, AMTR8, and TmR1b—demonstrated high inhibition, generally >30% inhibition. Differences in mycelial inhibition levels by these bacterial strains were statistically significant (*p* ≤ 0.05) (Table 1).

For *N. parvum* PUCV 1547, inhibition rates were 12% at 10 °C, 36% at 22 °C, and 37% at 30 °C. Inhibition was notably higher with *Pseudomonas* sp. strains TmR1b, AMCR2b, AMTR8, and GcR15a. In the case of *N. parvum* PUCV 1557, inhibition rates were 16% at 10 °C, 35% at 22 °C, and 36% at 30 °C, with significantly higher levels of inhibition with *Pseudomonas* sp. strains TmR1b, AMCR2b, AMTR8, and GcR15a. For *N. parvum* PUCV 1560, inhibition rates were 16% at 10 °C, 31% at 22 °C, and 52% at 30 °C, with higher inhibition levels observed with *Pseudomonas* sp. strains TmR1b, AMCR2b, AMTR8, and GcR15a. No significant inhibition by the other bacterial strains compared to the controls were observed.

*Pseudomonas* sp. strains TmR1b, AMCR2b, AMTR8, and GcR15a, which showed the highest biocontrol potential using in vitro dual antagonism assays, were selected for further in vitro and in vivo biocontrol assays.

Superscript letters denote statistically significant differences compared to the control at the corresponding temperature (Tukey’s test, *p* ≤ 0.05).

### 2.2. In Vitro Biocontrol Agar Plug Diffusion Assays for Grapevine Trunk Pathogens

**Figure 1:**
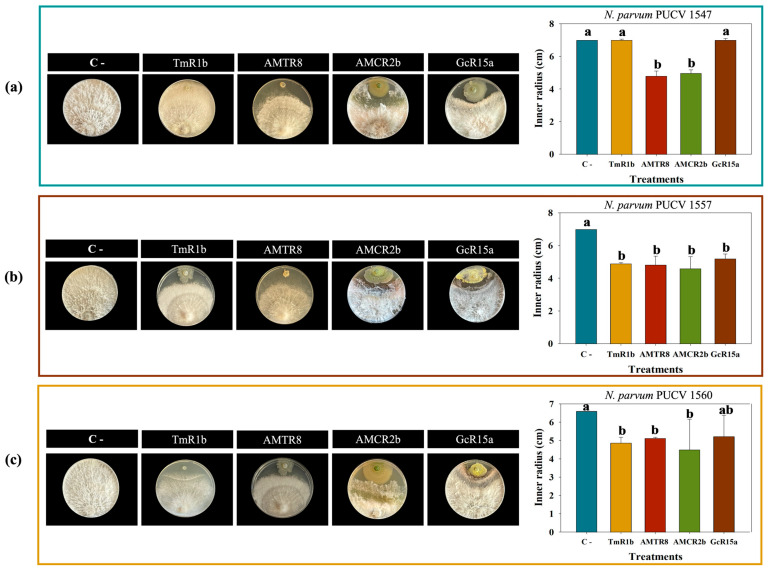
Biocontrol effects of native bacteria on the colony inner radius of *N. parvum* strains after 7 days at 22 °C, determined using the agar plug diffusion method. Effects on *N. parvum* strains: (**a**) PUCV 1547, (**b**) PUCV 1557, and (**c**) PUCV 1560. Data are presented as mean ± standard deviation (n = 4). Statistical significance was determined using Tukey’s test (*p* ≤ 0.05). Different letters indicate statistically significant differences between treatments. Abbreviations: “C−”, negative control; TmR1b, *Pseudomonas* sp. TmR1b; AMTR8, *Pseudomonas* sp. AMTR8; AMCR2b, *Pseudomonas* sp. AMCR2b; GcR15a, *Pseudomonas* sp. GcR15a.

Biocontrol agar plug diffusion assays indicated that the selected *Pseudomonas* sp. strains GcR15a, AMCR2b, AMTR8, and TmR1b significantly reduced (*p* ≤ 0.05) the mycelial growth of *N. parvum* strains PUCV 1547, PUCV 1557, and PUCV 1560 after seven days incubation at various temperatures (Figure 1, Figures S1–S3 and Table 2(a)).

Figure S1 shows the biocontrol effects at 10 °C. At 10 °C, fungal growth was slower than at higher temperatures. The most significant effects were observed by strain AMTR8 on *N. parvum* PUCV 1547 with 16% inhibition after 7 days, strain GcR15a on PUCV 1557 with 7% inhibition, and strain TmR1b on PUCV 1560 with 7% inhibition (Table 2(a)). At 15 °C (Figure S2), strain AMCR2b inhibited *N. parvum* PUCV 1547 (40%) and PUCV 1557 (14%) after 7 days, whereas strain GcR15a inhibited (25%) *N. parvum* PUCV 1560 (Table 2(a)).

The optimum temperature for *N. parvum* growth was 22 °C (Figure 1). Biocontrol effects on the fungus were significant after 7 days. At 22 °C, strain AMCR2b showed the highest inhibition of all strains, with 34% inhibition on PUCV 1547, 34% on PUCV 1557, and 33% on PUCV 1560 (Table 2(a)). At 30 °C, significant fungal inhibition was observed after 7 days (Figure S3). Strain AMCR2b inhibited PUCV 1547 (40%), PUCV 1557 (50%), and PUCV 1560 (45%) (Table 2(a)).

In negative controls, *N. parvum* strains showed the highest growth at 22 °C and 30 °C, with decreasing growth at 15 °C and 10 °C (Figure 1 and Figures S1–S3). Additionally, a noticeable change in the color of all *N. parvum* isolates was observed with BCA strains at 22 °C and 30 °C (Figure 1 and Figure S4).

### 2.3. In Vitro Biocontrol Double Plate Assays for Grapevine Trunk Pathogens

**Figure 2:**
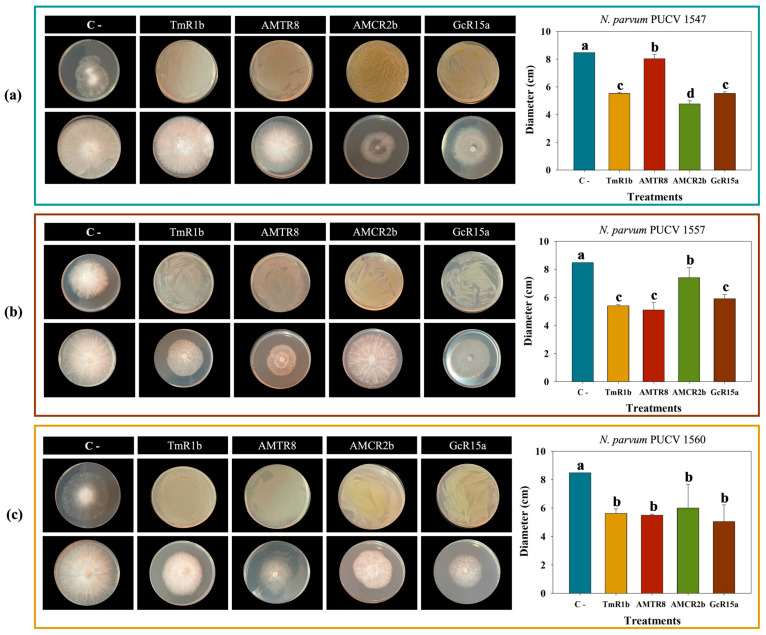
Biocontrol effects of native bacteria on *N. parvum* strains PUCV 1547, PUCV 1557, and PUCV 1560 after 72 h at 22 °C, determined by double plate method. The fungal colony diameter was measured. Effects of the native bacteria against *N. parvum* strains: (**a**) PUCV 1547, (**b**) PUCV 1557, and (**c**) PUCV 1560. Data are presented as mean ± standard deviation (n = 4). Statistical significance was determined using Tukey’s test (*p* ≤ 0.05). Different letters indicate statistically significant differences between treatments. Abbreviations: C−, negative control; TmR1b, *Pseudomonas* sp. TmR1b; AMTR8, *Pseudomonas* sp. AMTR8; AMCR2b, *Pseudomonas* sp. AMCR2b; GcR15a, *Pseudomonas* sp. GcR15a.

*N. parvum* strains exhibited higher growth at 22 °C and 30 °C, followed by 15 °C and 10 °C (negative control of Figure 2 and Figures S4–S6). Biocontrol double plate assays showed that the selected *Pseudomonas* sp. strains GcR15a, AMCR2b, AMTR8, and TmR1b significantly reduced (*p* ≤ 0.05) the mycelial growth of *N. parvum* strains PUCV 1547, PUCV 1557, and PUCV 1560 after 72 h at different temperatures (Figure 2 and Figures S4–S6, Table 2(b)).

At 10 °C, lower fungal growth was observed compared to growth at higher temperatures. In double plate assays, PGPB AMTR8 and TmR1b showed significant fungal growth reduction at 72 h compared to the control (Figure S4). Strain AMTR8 reached 30% inhibition of *N. parvum* strain PUCV 1560, whereas strain TmR1b achieved 25% inhibition of *N. parvum* strain PUCV 1547 and 36% inhibition of *N. parvum* strain PUCV 1557 (Table 2(b)).

At 15 °C, fungal growth was higher than at 10 °C. PGPB caused a relevant decrease in fungal growth after 72 h compared to the control (Figure S5). Bacterial strain AMTR8 significantly inhibited *N. parvum* PUCV 1560 (30%), whereas strains GcR15a strongly inhibited *N. parvum* strain PUCV 1547 (46%) and slightly inhibited *N. parvum* strain PUCV 1557 (5%) (Table 2(b)).

The highest growth of the fungus *N. parvum* was observed at 22 °C. Bacterial strains caused a significant fungal growth reduction at this temperature 72 h (Figure 2). Strain AMTR8 inhibited *N. parvum* strain PUCV 1557 (39%) and *N. parvum* strain PUCV 1560 (30%), whereas strain AMCR2b inhibited (43%) *N. parvum* strain PUCV 1547 (Table 2(b)).

At 30 °C, PGPB caused a significant decrease in fungal growth after 72 h (Figure S6). Strain AMCR2b inhibited *N. parvum* strain PUCV 1547 (26%) and *N. parvum* PUCV 1560 (22%), whereas strain GcR15a inhibited (13%) *N. parvum* PUCV 1557 (Table 2(b)).

### 2.4. In Vivo Test on Cuttings of Different Ages

The impact of three *N. parvum* strain on grapevine cuttings of different ages (1, 4, 5, and 25 years) previously inoculated with the selected four PGPB at temperatures of 10 °C, 22 °C, and 30 °C was studied.

**Figure 3:**
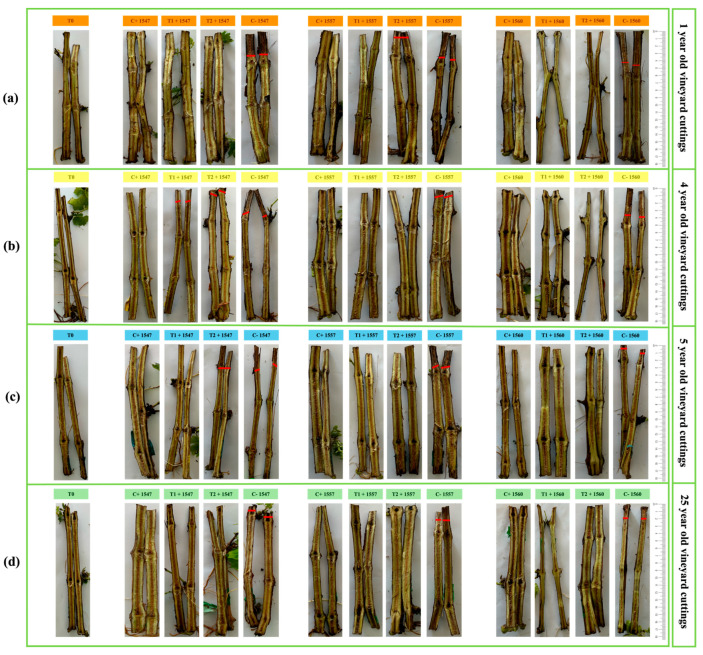
Severity of vascular lesions caused by *N. parvum* strains PUCV 1547, PUCV 1557, and PUCV 1560 in *V. vinifera* cv. Cabernet Sauvignon cuttings pre-inoculated with PGPB at 22 °C. The red line indicates how far the fungus grew. Effects of native bacteria pre-inoculation on vascular lesion length on *N. parvum* inoculated vineyard cuttings of (**a**) 1-year, (**b**) 4-years, (**c**) 5-years, and (**d**) 25-years. Abbreviations: T0, control treatment; C+, positive control (tebuconazole); T1, AMCR2b (*Pseudomonas* sp. AMCR2b); T2, GcR15a (*Pseudomonas* sp. GcR15a); C−, negative control (distilled water).

At 22 °C, grapevine cuttings of lower ages (1 and 4 years) exhibited larger lesions than older cuttings of 5 and 25 years (Table 2(c), Table S1, and Figure 3). In cuttings of vineyards 5 years old, shorter lesions (1 cm) were observed compared to younger cuttings (1.5 cm), highlighting the faster progression of *N. parvum* in younger wood tissues (Figure 3a–c). The 25-year-old grapevine cuttings showed mild lesions (1 cm) only in negative control, indicating higher resistance to *N. parvum*. *N. parvum* strain PUCV 1560 induced larger vascular lesions in several grapevine cuttings than strains PUCV 1547 and PUCV 1557 (Table S1). In contrast, no damage was observed in the non-inoculated cuttings, confirming the pathogenicity of the three *N. parvum* strains in *V. vinifera* cv. However, Cabernet Sauvignon cuttings showed a low infection rate (Figure 3).

Bacterial strains demonstrated biocontrol effects across all treatments, with inhibition ranging from 30–90% compared to the negative control (Table 2(c)). In addition, PGPB exhibited growth promotion effects in cuttings of different ages (Figure 3). In the presence of bacterial strain AMCR2b, significant reductions (71–90%) in vascular lesion length were observed in cuttings of all ages inoculated with *N. parvum* PUCV 1547 and PUCV 1560 (Figure 3, Table 2(c)). Bacterial strain GcR15a reduced vascular lesion length (53–88%) caused by *N. parvum* 1557 (Table 2(c)).

At 10 °C, no vascular injury was detected in cuttings from vineyards of 1, 4, 5, and 25 years inoculated with *N. parvum* strains PUCV 1547, PUCV 1557, and PUCV 1560. At 30 °C, cuttings of vineyards aged 1, 3, 4, and 25 years inoculated with *N. parvum* strains PUCV 1547, PUCV 1557, and PUCV 1560 showed evidence of microbial presence and rot in all treatments.

Vineyard cuttings of 1, 4, 5, and 25 years treated with tebuconazole remained healthy, showing 100% inhibition of *N. parvum* strains.

Figure S7 illustrates the growth promotion potential of vineyard cutting by *Pseudomonas* sp. strains AMCR2b and GcR15a compared to treatments without bacterial inoculation. Figure S7 shows that grapevine cutting treated with the pathogen exhibit reduced growth. In contrast, Figure S7b,c depicts cuttings treated with the pathogen and inoculated with strains AMCR2b and GcR15a, where increased leaf number and improved overall vineyard cuttings growth were observed. These results indicate the beneficial effects of *Pseudomonas* sp. strains AMCR2b and GcR15a on grapevine development, despite the presence of the pathogen *N. parvum*. Therefore, *Pseudomonas* sp. strains AMCR2b and GcR15a were selected for further in situ assays.

### 2.5. In Situ Biocontrol Test on Cuttings

For the in situ biocontrol test on cuttings assays, *Pseudomonas* sp. strains AMCR2b and GcR15a were selected based on the results observed in the in vitro and in vivo assays. *Rhodococcus* sp. strain PU4 was selected for in situ biocontrol test on cuttings based on the results previously reported by Larach and Vega-Celedón et al. [53].

**Figure 4:**
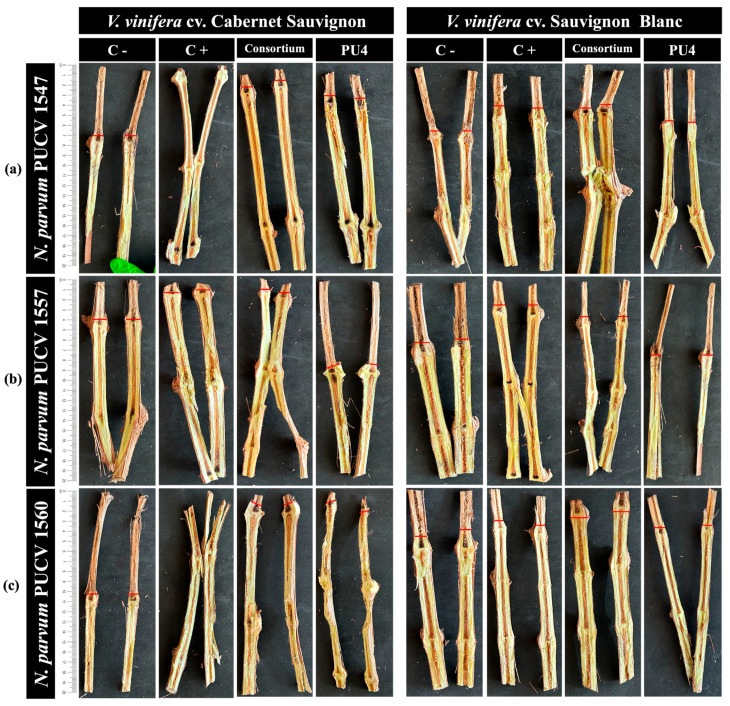
Severity of damage caused by *N. parvum* strains in 7-year-old vineyard shoots of *V. vinifera* cv. Cabernet Sauvignon and Sauvignon Blanc, pre-inoculated with native bacteria for biocontrol. The red line indicates how far the fungus grew. Effects of pre-inoculation with native bacteria on the vascular lesion length in shoots of *V. vinifera* cv. Cabernet Sauvignon and Sauvignon Blanc cuttings inoculated with *N. parvum* strains (**a**) PUCV 1547, (**b**) PUCV 1557, and (**c**) PUCV 1560. Abbreviations: C-, negative control (distilled water); C+, positive control (tebuconazole); Bacterial consortium: AMCR2b (*Pseudomonas* sp. AMCR2b) and GcR15a (*Pseudomonas* sp. GcR15a); PU4, *Rhodococcus* sp. PU4.

In the field in situ biocontrol test, the efficacy of various *N. parvum* strains on one-year-old Cabernet Sauvignon and Sauvignon Blanc grapevine cuttings, previously inoculated with a consortium of bacterial strains GcR15a and AMCR2b or the endophytic bacterial strain PU4, was evaluated after 150 days. Figure 4 illustrates that the bacterial strains, especially the consortium of strains GcR15a and AMCR2b, demonstrated significant biocontrol effects against different strains of *N. parvum* in Cabernet Sauvignon and Sauvignon Blanc cuttings.

Sauvignon Blanc cuttings were significantly more susceptible than Cabernet Sauvignon cuttings to *N. parvum* inoculation. Vascular lesion lengths in Sauvignon Blanc cuttings were up to 12 cm, compared to 8 cm in Cabernet Sauvignon cuttings (Table S2). The pathogen recovered from the lesions on the arms and identified morphologically as *N. parvum*.

Bacterial inoculation on Cabernet Sauvignon cuttings showed a fungal inhibition between 26–89% (Table 2(d)). Higher fungal inhibition by the consortium than by strain PU4 was observed. *N. parvum* strain PUCV 1560 strain caused larger vascular lesions than the other *N. parvum* strains (Figure 4, Table 2(d) and Table S2). Bacterial application on Sauvignon Blanc cuttings showed a fungal inhibition in the range 29–80% (Table 2(d)); the consortium showed higher inhibition than strain PU4. *N. parvum* strain PUCV 1547 caused the largest vascular lesions on Sauvignon Blanc cuttings (Figure 4). Seven-year-old vineyard shoots treated with tebuconazole remained healthy, depicting 100% inhibition of *N. parvum* strains.

## 3. Discussion

*N. parvum* is a prevalent and aggressive fungal pathogen of grapevines, which affects the permanent woody structures, including arms and trunks, causing GTD. The growing demand for environmentally friendly practices in viticulture [73,74] promotes the search for biocontrol agents against the fungi (e.g., *N. parvum*, *D. seriata*) associated to Botryosphaeria dieback in grapevines [35,46,53]. This study contributes to the development of biocontrol strategies based on Chilean native bacteria to mitigate the impact of *N. parvum* on viticulture.

Environmental temperature plays a critical role in the growth kinetics of the fungal phytopathogen *N. parvum* that causes GTD and the efficacy of biocontrol agents. It has been reported that conidia release under field conditions occurred at average weekly median temperatures above 10 °C [75]. *Botryosphaeriaceae* species disperse across a wide range of temperature, from 3 °C to 10 °C [48,76,77]. *Neofusicoccum* spp. grow within a temperature range of 10 °C to 35 °C, with optimal mycelial growth at 25 °C [78]. This underscores the importance of evaluating fungal pathogen behavior at 10 °C. In the present study, optimal radial growth of Chilean *N. parvum* strains was observed at 22 °C. Previous studies have described that the optimal growth temperature for *N. parvum* is in the range from 22 °C to 25 °C [50,79]. The reduced growth of Chilean *N. parvum* strains at more extreme temperatures (10 °C and 35 °C) reflects its adaptability to moderate climate, which may explain its high prevalence in vineyards of Central Chile during the grapevine growing season.

In this study, fifteen bacterial strains isolated from native wild flora and grapevine plants grown in various ecosystems in Chile [52,53] were evaluated as biocontrol agents. In previous studies, twelve of these bacterial strains demonstrated psychrotolerance, auxin production, phosphate solubilization, the presence of *nifH* (nitrogenase reductase) and *acdS* (1-aminocyclopropane-1-carboxylate (ACC) deaminase) genes, as well as anti-phytopathogenic activities [52,80]. These bacterial strains were tested for their potential as biocontrol agents against *N. parvum*, a fungal pathogen associated with GTDs [35]. *Pseudomonas* sp. strains AMCR2b, GcR15a, AMTR8, and TmR1b, which were isolated from wild flora in cold ecosystems, demonstrated in vitro significant antagonistic activity by inhibiting the radial growth of *N. parvum* across different temperatures (10 °C, 15 °C, 22 °C, and 30 °C) using dual-culture trials (Figure 1, Figure 2, Figures S1–S6 and Table 1). This study and previous reports [52,54,55,59,81,82,83,84,85,86] showed that the exploration of the biodiversity of diverse ecosystems in Chile is useful to isolate and select beneficial microorganisms for the agriculture (biocontrol of phytopathogens and plant growth promotion) and other biotechnological applications (e.g., bioremediation, antibiotic discovery). The results of our study underscore the versatility and potential of native bacteria from wild flora and especially these native psychrotolerant *Pseudomonas* strains to be used as effective biocontrol agents under a wide range of temperatures.

*Pseudomonas* sp. strains AMCR2b and GcR15a were the most effective bacterial strains for in vitro biocontrol of *N. parvum*, as revealed by agar plug diffusion and double-plate assays. *Pseudomonas* sp. strain AMCR2b achieved inhibition rates of up to 50% in agar diffusion tests and 43% in double-plate assays at 30 °C and 22 °C, respectively. *Pseudomonas* sp. strain GcR15a showed similar inhibitory effects, with inhibition of up to 46% at 15 °C (Table 2(a)). These results are in accordance with previous studies that described antimicrobial activities of *Pseudomonas* sp. strains AMCR2b and GcR15a against the phytopathogenic bacteria *P. syringae* pv. *syringae*, *Pectobacterium carotovorum*, and *Clavibacter michiganensis* subsp. *michiganensis* [52,80], and against the GTD associated fungus *D. seriata* [53]. Various *Pseudomonas* strains have been described as biocontrol agents. For example, *Pseudomonas putida* controls root pathogens such as *Ralstonia solanacearum* and *Verticillium dahliae* [87], and *Pseudomonas fluorescens* is able to suppress *Pythium ultimum* and *Phytophthora infestans* [88]. *Pseudomonas* sp. SH-C52 shows biocontrol activities [89]. Additionally, *P. chlororaphis* inhibits *Rosellinia necatrix* [90], and *P. protegens* CHA0 exhibits strong antifungal activity against *Fusarium* spp., *Phytophthora infestans*, and *Botrytis cinerea* [91]. Moreover, *P. aeruginosa* FG106 has antagonistic effects against *Alternaria alternata* and *Colletotrichum gloeosporioides* [92]. Inhibition of up to 22% of *N. parvum* by grapevine *Pseudomonas* endophytes has been reported [56]. The ability of *Pseudomonas* spp. to inhibit fungal growth through the production of diffusible and volatile organic compounds reveal their robust antifungal potential and suggests its applicability in field settings.

Based on their high biocontrol activities observed in vitro, *Pseudomonas* sp. AMCR2b and GcR15a were selected for in vivo tests using grapevine cuttings of varying ages and in situ experiments in *V. vinifera* vineyards. The trials with grapevine cuttings of different ages revealed important insights into the susceptibility of vineyard tissues to *N. parvum*. Young grapevine cuttings (1–5 years old) were more susceptible, with vascular lesion lengths reaching up to 5 cm, especially in the 1-year-old cuttings (Figure 3). This issue highlights the pathogen’s preference for young, actively growing tissues. However, the biocontrol of *N. parvum* by bacterial strains AMCR2b and GcR15a was observed across all grapevine cutting ages, achieving high inhibition (69–90%). These findings highlight the versatility of *Pseudomonas* strains as biocontrol agents, demonstrating their capability to protect both young and mature vineyards, an essential trait due to the diverse age composition of vineyard blocks. Our study is the first report on biocontrol trials on vineyard cuttings of different ages. It has been described that *Pseudomonas poae* strain BCA17 reduces lesion length and host colonization by *N. luteum* [93]. Strain BCA17 suppressed *N. luteum in planta* using both cuttings and potted grapevines. Our biocontrol trials indicate the potential of *Pseudomonas* strains in managing grapevine trunk diseases caused by *N. parvum*.

In this study, field trials demonstrated larger lesions on shoots (Figure 4) compared to cuttings (Figure 3), suggesting that the natural conditions of the field may enhance the virulence of *N. parvum*. This observation aligns with the pathogen’s ability to surpass host defenses by producing phytotoxins that increase its aggressiveness [94,95]. Although plants affected by this pathogen may show symptoms in the field only one or two years after infection [79], we observed *N. parvum* infection in all our trials, demonstrating its adaptation to different environmental conditions. A wide diversity of phytotoxins produced by *N. parvum* has been reported [94], which allowed the fungus to adapt to different environmental conditions. The inhibitory effects of *Pseudomonas* strains highlight their potential to suppress fungal spread even in challenging environments.

The observed effectiveness of *Pseudomonas* sp. strains AMCR2b and GcR15a to control *N. parvum* is in accordance with other studies that report the biocontrol capabilities of *Pseudomonas* species. *Pseudomonas* is a genus well-known for its ecological adaptability, fast growth, and remarkable capability to antagonize plant pathogens and protect plants from abiotic stresses that are intensified by climate change [52,53,96,97]. *P. poae* BCA17 colonizes grapevine tissues, suppresses fungal pathogens, and reduces in vivo lesion lengths [98]. *Pseudomonas* spp. reduces host colonization and lesion lengths caused by fungal infections [93]. *Pseudomonas* species have been shown to produce hydrolytic enzymes, antibiotics, and secondary metabolites that target fungal pathogens [99,100]. The biocontrol by *Pseudomonas* are largely attributed to the production of a diverse array of bioactive metabolites, including volatile organic compounds [101,102], siderophores [103], phenazines [104], antibiotics [105,106], and non-ribosomal peptide synthetase products that inhibit pathogen growth [107,108,109,110]. These mechanisms may explain the observed inhibitory effects of the *Pseudomonas* strains on *N. parvum* isolates.

This study demonstrates the biocontrol potential against the fungus *N. parvum* of *Pseudomonas* sp. strains AMCR2b and GcR15a, which are psychrotolerant native bacteria from wild flora in cold ecosystems in Chile. These strains showed significant inhibition of fungal growth across various temperatures and under diverse experimental conditions. By addressing the challenges of modern agriculture, this report contributes to the development of sustainable disease management strategies that reduce the reliance on chemical fungicides. Future research will focus on the identification of the specific bioactive compounds involved in biocontrol of the phytopathogen *N. parvum*, the optimization of their application in field conditions, and the exploration of their interactions with host plants and environmental factors. This study aims to promote the application of effective and sustainable biocontrol approaches for managing grapevine trunk diseases in viticulture.

## 4. Materials and Methods

### 4.1. Chemicals, Reagents, and Culture Media

*D*-Glucose was obtained from Merck (Darmstadt, Germany). Yeast extract and Bacto proteose peptone No. 3 were purchased from Difco Laboratories (Franklin Lakes, NJ, USA). Malt extract and potato dextrose agar (PDA) were obtained from HiMedia Laboratories (West Chester, PA, USA).

### 4.2. Microorganisms and Vegetable Materials of This Study

The beneficial psychrotolerant bacteria used for the biocontrol studies were *Pseudomonas* sp. TmR1b, *Pseudomonas* sp. NUR4a, *Pseudomonas* sp. AMCR2b, *Pseudomonas* sp. TmR5a, *Brachybacterium* sp. TmP30, *Pseudomonas* sp. TmR7, *Pseudomonas* sp. AMTR8, *Pseudomonas* sp. GcR15a, *Frondihabitans* sp. GpP26d, *Curtobacterium* sp. BmP22c, and *Arthrobacter* sp. BmP28 was isolated from native flora by Vega-Celedón et al. [52,80] and the endophytic bacteria *Bacillus* sp. PU3, *Rhodococcus* sp. PU4, and *Staphylococcus* sp. PU18 were isolated from ‘Cabernet Sauvignon’ non-grafted vineyards (Table 3) by Larach et al. [35]. *Pseudomonas protegens* CHA0 was used as a reference biocontrol strain [52,91]. Three phytopathogenic strains of the fungus *N. parvum* were used in this study: PUCV 1547, PUCV 1557, and PUCV 1560 (Table 4), which were isolated by Larach et al. [35]. These microorganisms were obtained from the culture collections of the Molecular Microbiology and Environmental Biotechnology Laboratory (Universidad Técnica Federico Santa María, Valparaíso, Chile) and the Faculty of Agronomy (Pontificia Universidad Católica de Valparaíso, Quillota, Chile). The cuttings were acquired from the Maule Region 35°00′30.1″ S 71°24′08.7″ W and the field trials were carried out in the vineyard of the La Palma Experimental Station 32°53′43.5″ S 71°12′07.5″ W of the Pontificia Universidad Católica de Valparaíso (PUCV).

### 4.3. In Vitro Analysis of Biocontrol by Bacteria

#### 4.3.1. Dual Antagonism Assays of Bacterial Strains Against Grapevine Trunk Pathogens

The antimicrobial activity of the 15 bacterial strains against the phytopathogenic fungi *N. parvum* strains PUCV 1547, PUCV 1557, and PUCV 1560 was carried out using the dual culture method according to Begum et al. [111], with modifications. Previously, the bacteria were cultured in yeast malt (YM; 10 g L^−^^1^ of glucose, 3 g L^−^^1^ of malt extract, 5 g L^−^^1^ of peptone, 3 g L^−^^1^ of yeast extract) for 72 h at 22 °C. The phytopathogenic fungi were cultivated in potato dextrose agar acidified with 0.5 mL of 96% lactic acid (APDA) medium for 5 days in the dark at 28 °C. One cm from the edge of a PDA (Himedia) plate, an agar disk (0.5 cm) of bacteria was seeded, and in the opposite direction, an agar disk of mycelium of the phytopathogenic fungus with a sterilized punch. The treatments consisted of confronting each bacterium against each pathogenic fungus. As an adverse control treatment, only an agar disk with fungal mycelium and an agar disk without bacteria were used and evaluated twice with four replicates of each treatment. The plates were incubated for 7 days at 10 °C, 22 °C, and 30 °C. The growth of the fungus in the inner radius was recorded after 3, 5, and 7 days.

#### 4.3.2. In Vitro Biocontrol Assays Using Agar Plug Diffusion for Grapevine Trunk Pathogens

The effects of biocontrol on the *N. parvum* strains were evaluated using the agar plug diffusion method as described by Olivera et al. [59], with modifications. For pathogenic fungi, *N. parvum* strains PUCV 1547, PUCV 1557, and PUCV 1560 were grown in Petri dishes with APDA medium by seeding an agar disk with actively growing mycelium for 5 days in the dark at 28 °C. Biocontrol agents (bacterial strains GcR15a, AMCR2b, AMTR8, and TmR1b) were grown in Petri dishes in YM medium for ~12 h and then adjusted to turbidity at 600 nm of 1 using a spectrophotometer (BOECO S-300, Hamburg, Germany). Subsequently, 100 µL of each isolate were deposited on each plate, allowed to dry, and further incubated for 72 h at room temperature.

One cm from the edge of a PDA plate (maximum radius of 9 cm), a 0.5 cm disk of bacteria was seeded, and in the opposite direction, a disk of the mycelium of the phytopathogenic fungus was placed using a sterilized punch. As a negative control treatment, only the fungus was used without the bacteria, and it was evaluated twice with four replicates for each treatment. The plates were incubated for 7 days at 10 °C, 15 °C, 22 °C, and 30 °C. The experiment was evaluated at 3, 5, and 7 days. Photographs of the plates were taken, and the internal radius of the mycelium was measured. The inhibition percentage of each treatment was calculated using the Equation (1) [112].

(1) $$\mathrm{Percentage}\;\mathrm{of}\;\mathrm{inhibition}\;(\%)=\left(\frac{\left(R-r\right)}{R}\right)\times 100$$

where *R* and *r* are the radii of fungal growth toward the control and toward the bacteria, respectively [112].

#### 4.3.3. In Vitro Biocontrol Assays Double Plate Method for Grapevine Trunk Pathogens

The effect of biocontrol on the *N. parvum* isolates was carried out using the double plate method, as reported by Delgado et al. [113], with modifications. For pathogenic fungi, *N. parvum* strains PUCV 1547, PUCV 1557, and PUCV 1560 were grown in APDA medium in Petri dishes by seeding an agar disk with actively growing mycelium for 5 days in the dark at 28 °C. Biocontrol agents (bacterial strains GcR15a, AMCR2b, AMTR8, and TmR1b) were grown in YM medium in Petri dishes for ~12 h and then adjusted to turbidity at 600 nm of 1 using a spectrophotometer (BOECO S-300, Hamburg, Germany). Subsequently, 100 µL of each isolate were deposited on each plate, allowed to dry and further incubated for 72 h at room temperature.

The bacteria were placed in streaks on YM medium in a Petri dish and on PDA medium in another Petri dish (both with a 9 cm radius), in the center of which a disc (0.5 cm) of the mycelium of the *N. parvum* isolates was planted; both plates were sealed with parafilm. The fungus without bacteria was used as a negative control treatment; it was repeated twice with four replicates for each treatment. The plates were incubated for 3 days at 10 °C, 15 °C, 22 °C, and 30 °C. The experiment was evaluated after 24, 48, and 72 h. Photographs of the plates were taken, and the diameter of the mycelium growth was recorded. The inhibition percentage of each treatment was calculated using the Equation (1).

### 4.4. In Vivo Biocontrol by BCAs

#### 4.4.1. Inoculations of Fungi and Bacteria

Spore suspensions were prepared following the methodology described by Larach et al. [35]. An agar disk with 5-day-old mycelium of each isolate was placed in Petri dishes containing 2% agar water and autoclaved pine needles. The plates were incubated in a room-temperature chamber under near-ultraviolet light (λ = 320 nm) until pycnidia production and conidia development. The mature pycnidia were ground in sterile distilled water, and the solution was filtered through cheesecloth. A suspension of 1 × 10^4^ conidia µL^−1^ was inoculated into each freshly cut tissue.

The bacteria solution was prepared as described by Vega-Celedón et al. [52]. The bacteria were grown in YM medium for 24 h at room temperature. Bacteria grown on the plates were collected and placed in sterile 50 mL Falcon tubes with 30 mL of YM medium. The turbidity was measured at 600 nm and adjusted to 0.2 by diluting with sterile distilled water, obtaining a final bacterial concentration of 1 × 10^8^ CFU mL^−1^.

#### 4.4.2. In Vivo Test on Cuttings

Bacterial biocontrol against pathogenic fungi was determined in cuttings following the methodology of Haidar et al. [114] and Kotze [69]. From a vineyard of the Cabernet Sauvignon variety, semi-lignified cuttings of the year were isolated from rows of vineyards of different ages 1, 4, 5, and 25 years. Cuttings 18 cm long were taken, disinfected with 1% sodium hypochlorite for 5 min and 95% ethanol for 30 s, and washed three times with sterile distilled water (SDW). Finally, the cuttings were dried at room temperature inside a laminar flow chamber. The cuttings were kept at 5 °C for two weeks before use. A fresh wound was made and immediately inoculated with 50 µL of each bacterial suspension in YM medium, YM medium (negative control, C−), and tebuconazole (0.5% *wv*^−1^, positive control, C+). After application of the treatments, the cuttings were left at room temperature until complete uptake of the inoculum. Each section was then placed in a humid chamber with a disinfected plastic rack that included a moistened absorbent paper (5 mL of SDW) and placed at 10 °C, 22 °C, and 30 °C. After 24 h, the cuttings were inoculated with 50 µL of *N. parvum* suspension at the same end where the previous inoculation was performed. Cuttings inoculated with each *N. parvum* isolate and its treatments (GcR15a, AMCR2b, C−, and C+) were placed in humid chambers at 10 °C, 22 °C, and 30 °C, with six replicate per treatment (n = 6). The assay was evaluated 120 days after inoculation with the pathogen. The length of the vascular lesions was measured in each detached section. Each treatment’s inhibition percentage was calculated using Equation (1).

#### 4.4.3. In Situ Biocontrol Test on Cuttings

The experiment was carried out in cv. Cabernet Sauvignon and Sauvignon Blanc plants in an experimental vineyard at La Palma Experimental Station of PUCV. The selected vineyard did not present previous GTD symptoms and was managed in a bilateral cordon system, with spur pruning to 4 buds. The inoculations were carried out on one-year-old shoots, and the cut was made at the height of 4 buds; they were immediately inoculated with 50 µL of each bacterial suspension in YM medium, YM medium (negative control, C−), and tebuconazole (0.5% *wv*^−1^, positive control, C+). After 24 h, the cuttings were inoculated with 50 µL of *N. parvum* suspension at the same end where the previous inoculation was performed. The cuttings were inoculated with each *N. parvum* isolate and subjected to its treatments (consortium of strains GcR15a and AMCR2b, endophytic strain PU4, C−, and C+).

#### 4.4.4. Pathogen Damage Assessment and Recovery

The length of the vascular lesions was recorded 120 days after tissue inoculation with the fungus. The damage produced in each tissue was evaluated in the field, and the plant material was cut to measure the lesions. The assay was performed in duplicate. To recover the fungus, tissue samples were taken from the area of lesion progression, disinfected with 1% sodium hypochlorite for 5 s, washed three times in SDW, and cultured in APDA medium for complete Koch’s postulates. *N. parvum* was identified by morphological identified by examining conidial shape under a microscope, following the methodology described by Larach et al. [35].

### 4.5. Statistical Analysis

Data was analyzed using one-way analysis of variance (ANOVA) [115] to study the effects of biological control strains. To satisfy the assumptions of homogeneity of variance (Shapiro-Wilks test), when the assumptions were not met, a nonparametric analysis of variance was performed with the Kruskal-Wallis test. Data means were compared using the Tukey test to detect significant differences (*p* ≤ 0.05) [116] using Infostat software version 2017. Field trials were randomized entirely, and 5 replicates were used for each treatment.

## 5. Conclusions

In this study, Chilean native bacteria were evaluated as potential biocontrol agents against the wood fungus *N. parvum* that pose a significant threat to grapevine crops globally. Native PGPB *Pseudomonas* sp. strains AMCR2b and GcR15a, which were isolated from native flora, demonstrated the ability to inhibit the mycelial growth of *N. parvum* isolates across different temperatures. In addition, the study revealed that *N. parvum* is more aggressive in the tissues of young grapevines, highlighting the vulnerability of these plants. The experiments provided valuable insights into the interactions between the native bacteria and the fungus *N. parvum* in vitro, in vivo, and in situ on field. Future efforts should be focused on identifying the bioactive compounds produced by the bacterial strains. These results offer a promising sustainable approach to address the viticulture challenge of GTD, paving the way for the development of more effective disease biocontrol strategies.

## Figures and Tables

**Table 1:** First selection of Chilean native bacterial strains based on mycelial growth inhibition of *N. parvum* strains at different temperatures, determined using dual antagonism assays.

Strains	PUCV 1547			PUCV 1557			PUCV 1560		
	10 °C	22 °C	30 °C	10 °C	22 °C	30 °C	10 °C	22 °C	30 °C
C−	2.5 ± 0.2 ^abc^	7 ± 0.0 ^a^	7 ± 0.0 ^a^	2.1 ± 0.17 ^ab^	7 ± 0.0 ^a^	7 ± 0.0 ^a^	2.3 ± 0.15 ^ab^	7 ± 0.0 ^a^	7 ± 0.0 ^a^
*Pseudomonas* sp. TmR1b	2.25 ± 0.3 ^c^	3.2 ± 2.0 ^c^	5.9 ± 1.12 ^abc^	2 ± 0.14 ^ab^	5.22 ± 0.33 ^bc^	6.2 ± 0.6 ^a^	2.1 ± 0.15 ^ab^	2.87 ± 2.09 ^d^	2.77 ± 0.43 ^bcd^
*Pseudomonas* sp. AMTR8	2.2 ± 0.05 ^c^	5.1 ± 1.3 ^ab^	3.5 ± 1.04 ^d^	2.1 ± 0.1 ^ab^	4.4 ± 0.28 ^cd^	2.8 ± 0.33 ^b^	2.3 ± 0.1 ^ab^	4.9 ± 0.21 ^bc^	4.45 ± 0.33 ^d^
*Pseudomonas* sp. AMCR2b	2.5 ± 0.14 ^abc^	4.5 ± 0.5 ^bc^	3.46 ± 0.24 ^d^	2 ± 0.05 ^ab^	3.95 ± 0.05 ^d^	3.5 ± 0.21 ^b^	2.2 ± 0.15 ^ab^	3.6 ± 0.20 ^cd^	2.8 ± 0.67 e
*Pseudomonas* sp. GcR15a	2.27 ± 0.09 ^bc^	3.2 ± 0.99 ^c^	4.7 ± 0.56 ^bcd^	1.9 ± 0.05 ^ab^	4.5 ± 0.55 ^cd^	3.1 ± 0.7 ^b^	2.2 ± 0.24 ^ab^	4.7± 0.20 ^bc^	4.4 ± 0.86 ^d^
*Pseudomonas* sp. TmR5a	2.3 ± 0.28 ^bc^	6.7 ± 0.28 ^a^	7 ± 0.0 ^a^	1.8 ± 0.43 ^a^	5.9 ± 0.83 ^b^	6.6 ± 0.40 ^a^	1.9 ± 0.40 ^ab^	5.5 ± 0.95 ^ab^	7 ± 0.0 ^a^
*Brachybacterium* sp. TmP30	2 ± 0.20 ^c^	7 ± 0.0 ^a^	6.0 ± 0.72 ^ab^	2.1 ± 0.17 ^ab^	7 ± 0.0 ^a^	6.5 ± 0.9 ^a^	2.2 ± 0.26 ^ab^	7 ± 0.0 ^a^	7 ± 0.0 ^a^
*Pseudomonas* sp. TmR7	2.3 ± 0.14 ^bc^	6.36 ± 0.80 ^ab^	7 ± 0.0 ^a^	1.8 ± 0.09 ^ab^	7 ± 0.0 ^a^	5.8 ± 0.78 ^a^	1.7 ± 0.32 ^b^	7 ± 0.0 ^a^	5.9 ± 1.21 ^abc^
*Curtobacterium* sp. BmP22c	2.3 ± 0.14 ^bc^	7 ± 0.0 ^a^	7 ± 0.0 ^a^	2.1 ± 0.2 ^ab^	6.8 ± 0.4 ^a^	5.9 ± 1.18 ^a^	2.2 ± 0.22 ^ab^	7 ± 0.0 ^a^	6.0 ± 0.42 ^abc^
*Frondihabitans* sp. GpP26d	2.57 ± 0.15 ^abc^	7 ± 0.0 ^a^	6.9 ± 0.05 ^a^	2.1 ± 0.2 ^ab^	7 ± 0.0 ^a^	6.8 ± 0.4 ^a^	2.4 ± 0.24 ^a^	7 ± 0.0 ^a^	7 ± 0.0 ^a^
*Arthrobacter* sp. BmP28	2.32 ± 0.26 ^bc^	7 ± 0.0 ^a^	6.5 ± 0.85 ^a^	2.3 ± 0.23 ^a^	7 ± 0.0 ^a^	6.3 ± 0.47 ^a^	2.4 ± 0.15 ^a^	7 ± 0.0 ^a^	6.75 ± 0.5 ^ab^
*Pseudomonas* sp. NUR4a	2.6 ± 0.14 ^abc^	7 ± 0.0 ^a^	6.57 ± 0.56 ^a^	1.7 ± 0.24 ^b^	7 ± 0.0 ^a^	6.9 ± 0.17 ^a^	2.2 ± 0.15 ^ab^	5.27 ± 1.6 ^abc^	6.5 ± 0.67 ^abc^
*Bacillus* sp. PU3	3 ± 0.25 ^a^	7 ± 0.0 ^a^	7 ± 0.0 ^a^	1.9 ± 0.26 ^ab^	7 ± 0.0 ^a^	6.5 ± 0.86 ^a^	2 ± 0.25 ^ab^	7 ± 0.0 ^a^	6.7 ± 0.5 ^ab^
*Rhodococcus* sp. PU4	3 ± 0.28 ^a^	6.7 ± 0.6 ^a^	6.22 ± 0.60 ^a^	1.9 ± 0.23 ^ab^	7 ± 0.0 ^a^	7 ± 0.0 ^a^	2 ± 0.28 ^ab^	7 ± 0.0 ^a^	6.7 ± 0.57 ^ab^
*Staphylococcus* sp. PU18	2.8 ± 0.5 ^ab^	6.4 ± 0.35 ^a^	6.1 ± 0.98 ^ab^	1.9 ± 0.26 ^ab^	5.6 ± 0.23 ^b^	6.4 ± 0.4 ^a^	2 ± 0.25 ^ab^	6.5 ± 0.3 ^ab^	7 ± 0.0 ^a^
*Pseudomonas protegens* CHA0	2.5 ± 0.15 ^ab^	6.36 ± 3.01 ^ab^	4.4 ± 0.45 ^cd^	2.2 ± 0.25 ^a^	7 ± 0.0 ^a^	6 ± 0.28 ^a^	2.2 ± 0.25 ^ab^	7 ± 0.0 ^a^	5.2 ± 0.5 ^cd^

Data are expressed as mean ± standard deviation (n = 4); superscript letters indicate statistically significant differences compared to the control (Tukey’s test, *p* ≤ 0.05).

**Table 2:** Inhibition by native bacteria of mycelial growth of *N. parvum* strains using different assays. (a) In vitro inhibition (%) of *N. parvum* growth using the agar plug diffusion method at various temperatures. (b) In vitro inhibition (%) of *N. parvum* growth using the double plate method at various temperatures. (c) In vivo inhibition (%) of *N. parvum* growth in grapevine cuttings. (d) In situ inhibition (%) of *N. parvum* growth in grapevine shoots.

	Essays	Variety	T °C	Grapevine Age	*N. parvum* Strain	Inhibition (%) by Biocontrol Agents				
						C−	TmR1b	AMTR8	AMCR2b	GcR15a
(a)	Agar plug diffusion method	-	10 °C	-	PUCV 1547	0 ^e^	12 ^a^	16 ^a^	0 ^e^	13 ^a^
					PUCV 1557	0 ^e^	5 ^bc^	2 ^c^	4 ^cd^	7 ^b^
					PUCV 1560	0 ^e^	7 ^b^	0 ^e^	1 ^e^	4 ^c^
			15 °C	-	PUCV 1547	0 ^h^	12 ^b^	16 ^a^	0 ^h^	13 ^b^
					PUCV 1557	0 ^h^	5 ^bc^	2 ^gh^	4 ^d^	7 ^de^
					PUCV 1560	0 ^h^	7 ^fgh^	0 ^h^	1 ^de^	4 ^c^
			22 °C	-	PUCV 1547	0 ^f^	0 ^f^	31 ^ab^	34 ^a^	26 ^cd^
					PUCV 1557	0 ^f^	30 ^bc^	31 ^ab^	34 ^a^	26 ^de^
					PUCV 1560	0 ^f^	28 ^d^	24 ^d^	33 ^ab^	20 ^e^
			30 °C	-	PUCV 1547	0 ^i^	37 ^ef^	39 ^de^	40 ^cde^	10 ^h^
					PUCV 1557	0 ^i^	46 ^bc^	44 ^bcd^	50 ^a^	31 ^f^
					PUCV 1560	0 ^i^	34 ^f^	42 ^bcd^	45 ^b^	23 ^g^
(b)	Double plate method	-	10 °C	-	PUCV 1547	0 ^g^	25 ^cd^	16 ^e^	22 ^d^	9 ^f^
					PUCV 1557	0 ^g^	36 ^a^	0 ^g^	15 ^e^	32 ^ab^
					PUCV 1560	0 ^g^	27 ^cd^	30 ^bc^	22 ^d^	10 ^ef^
			15 °C	-	PUCV 1547	0 ^f^	34 ^b^	45 ^a^	30 ^bc^	46 ^a^
					PUCV 1557	0 ^f^	3 ^f^	0 ^f^	0 ^f^	5 ^e^
					PUCV 1560	0 ^f^	27 ^cd^	30 ^bcd^	22 ^d^	10 ^e^
			22 °C	-	PUCV 1547	0 ^g^	35 ^bc^	5 ^g^	43 ^a^	35 ^bc^
					PUCV 1557	0 ^g^	36 ^bc^	39 ^b^	11 ^f^	31 ^cd^
					PUCV 1560	0 ^g^	27 ^de^	30 ^de^	22 ^e^	10 ^f^
			30 °C	-	PUCV 1547	0 ^e^	0 ^e^	0 ^e^	26 ^a^	14 ^b^
					PUCV 1557	0 ^e^	0 ^e^	6 ^d^	8 ^cd^	13 ^bc^
					PUCV 1560	0 ^e^	12 ^bc^	9 ^cd^	22 ^a^	21 ^a^
(c)	Cuttings	*V.* *vinifera* cv. Cabernet Sauvignon	22 °C			C−		C+	AMCR2b	GcR15a
				1	PUCV 1547	0 ^c^		100 ^a^	90 ^a^	95 ^b^
				4		0 ^a^		100 ^a^	75 ^a^	55 ^a^
				5		0 ^a^		100 ^a^	95 ^a^	6 ^a^
				25		0 ^a^		100 ^a^	80 ^a^	90 ^a^
				1	PUCV 1557	0 ^c^		100 ^a^	95 ^a^	53 ^b^
				4		0 ^a^		100 ^a^	60 ^a^	70 ^a^
				5		0 ^a^		100 ^a^	81 ^a^	88 ^a^
				25		0 ^a^		100 ^a^	75 ^a^	84 ^a^
				1	PUCV 1560	0 ^c^		100 ^a^	80 ^ab^	50 ^b^
				4		0 ^a^		100 ^a^	90 ^a^	74 ^a^
				5		0 ^a^		100 ^a^	83 ^a^	42 ^a^
				25		0 ^a^		100 ^a^	84 ^a^	69 ^a^
(d)	Shoots	*V.* *vinifera* cv. Cabernet Sauvignon	-			C−	C+	PU4	AMCR2b–GcR15a	
				7	PUCV 1547	0 ^d^	91 ^a^	68 ^b^	82 ^c^	
					PUCV 1557	0 ^d^	83 ^a^	26 ^b^	74 ^c^	
					PUCV 1560	0 ^c^	94 ^a^	26 ^b^	89 ^b^	
		*V. vinifera* cv. Sauvignon Blanc	-	7	PUCV 1547	0 ^c^	83 ^a^	74 ^a^	80 ^b^	
					PUCV 1557	0 ^c^	97 ^a^	29 ^b^	30 ^b^	
					PUCV 1560	0 ^d^	18 ^c^	39 ^a^	76 ^b^	

Superscript letters indicate statistically significant differences compared to the control (Tukey’s test, *p* ≤ 0.05).

**Table 3:** Chilean native bacteria used for biocontrol of *N. parvum* in this study.

Native Bacteria	Locality, Region	Wild Plant	Closest Organism (Partial 16S rRNA Gene Sequence)	Identity (%)	Accession Number	Reference
*Pseudomonas* sp. TmR1b	Los Libertadores, Los Andes, Valparaíso	*Thlaspi* sp. (*R*)	*Pseudomonas azotoformans* strain 16d-S37	693/693 (100%)	MW548351	[52]
*Pseudomonas* sp. AMTR8	Los Libertadores, Los Andes, Valparaíso	*Thlaspi* sp. (*R*)	*Pseudomonas brassicacearum* strain DF41	657/657 (100%)	*	[80]
*Pseudomonas* sp. AMCR2b	Los Libertadores, Los Andes, Valparaíso	*Calycera* sp. (*R*)	*Pseudomonas asgharzadehiana* strain SWRI132	689/689 (100%)	*	[80]
*Pseudomonas* sp. GcR15a	Vicinity of El teniente Mine, Machalí, O’higgins	*Gnaphallium* sp. (*R*)	*Pseudomonas orientalis* strain R4-35-08	706/707 (99.86%)	MW548343	[52]
*Pseudomonas* sp. TmR5a	Los Libertadores, Los Andes, Valparaíso	*Thlaspi* sp. (*R*)	*Pseudomonas cedrina* strain K19B	722/722 (100%)	MW548356	[52]
*Brachybacterium* sp. TmP30	Los Libertadores, Los Andes, Valparaíso	*Thlaspi* sp. (*P*)	*Brachybacterium tyrofermentans* strain AFS097178	676/677 (99%)	MW548378	[52]
*Pseudomonas* sp. TmR7	Los Libertadores, Los Andes, Valparaíso	*Thlaspi* sp. (*R*)	*Pseudomonas syringae* pv. *actinidiae strain 18YN-PSA-C2*	693/695 (99%)	MW548359	[52]
*Curtobacterium* sp. BmP22c	Chabunco Park, Punta Arena, Magallanes and Chilean Antartica	*Berberis* sp. (*P*)	*Curtobacterium flaccumfaciens* pv. *flaccumfaciens* strain Cff1037	657/657 (100%)	MW548393	[52]
*Frondihabitans* sp. GpP26d	Shangri-La EcoPark, Las Trancas Valley, Ñuble	*Gaultheria* sp. (*P*)	*Frondihabitans sucicola* strain HP-S2	657/657 (100%)	MW548348	[52]
*Arthrobacter* sp. BmP28	Chabunco Park, Punta Arena, Magallanes and Chilean Antartica	*Berberis* sp. (*P*)	*Arthrobacter citreus* strain TTS-AB-A36	664/667 (99%)	MW548382	[52]
*Pseudomonas* sp. NUR4a	Chabunco Park, Punta Arena, Magallanes and Chilean Antartica	*Berberis* sp. (*P*)	*Pseudomonas baetica* strain IHB B 4123	688/694 (99%)	MW548343	[52]
*Bacillus* sp. PU3	Peralillo, O’Higgins	*Vitis vinifera* (*E*)	*Bacillus xiamenensis* strain INV FIR70	725/725 (100%)	*	This study
*Rhodococcus* sp. PU4	Peralillo, O’Higgins	*Vitis vinifera* (*E*)	*Rhodococcus qingshengii* strain H-cryo-48	682/685 (99.56%)	OQ244039	[53]
*Staphylococcus* sp. PU18	Peralillo, O’Higgins	*Vitis vinifera* (*E*)	*Staphylococcus epidermidis* strain VU-UCBMSH2	719/719 (100%)	*	This study

* No access available; (*R*): Rhizosphere; (*P*): Phyllosphere; (*E*): Endophyte.

**Table 4:** Phytopathogenic *N. parvum* strains used in this study.

*N. parvum* Strains	Locality, Region	Access No. GenBank		Reference
		ITS	BT	
PUCV 1547	Peralillo, O’Higgins	KM870224	KP762483	[35]
PUCV 1557	Palmilla, O’Higgins	KM870225	KP762484	[35]
PUCV 1560	Talca, Maule	KM870226	KP762485	[35]

ITS = internal transcribed spacer region. BT = β-tubulin gene.

## Data Availability

The original contributions presented in the study are included in the article/Supplementary Materials, further inquiries can be directed to the corresponding authors.

## Supplementary Materials

The following supporting information can be downloaded at: https://www.mdpi.com/article/10.3390/plants14071043/s1, Figures S1–S3. Biocontrol effects of native bacteria on the inner colony radius of *N. parvum* strains after 7 days, determined using the agar plug diffusion method. Figures S4–S6. Biocontrol effects of native bacteria on *N. parvum* strains after 72 h, determined using the double plate method. Fungal colony diameter was measured. Figure S7. Growth stimulation by *Pseudomonas* sp. strains AMCR2b and GcR15a at 22 °C of vineyard cuttings of 1, 4, 5, and 25 years treated with *N. parvum*. Table S1. Effects of native bacteria on vascular lesion lengths (cm) caused by *N. parvum* strains in vineyard cuttings of different ages. Table S2. Effects of native bacteria on vascular lesion lengths (cm) caused by *N. parvum* strains in 7-year-old vineyard shoots.
