# Supplementary material for: Native Bacteria Are Effective Biocontrol Agents at a Wide Range of Temperatures of Neofusicoccum parvum, Associated with Botryosphaeria Dieback on Grapevine
Source: Plants (Basel). 2025 Mar 27;14(7):1043. doi: 10.3390/plants14071043 (PMC11990564; doi:10.3390/plants14071043)
Supplement: Supplementary file 1 [file plants-14-01043-s001.zip › plants-3494103-supplementary.pdf]

Supplementary Materials

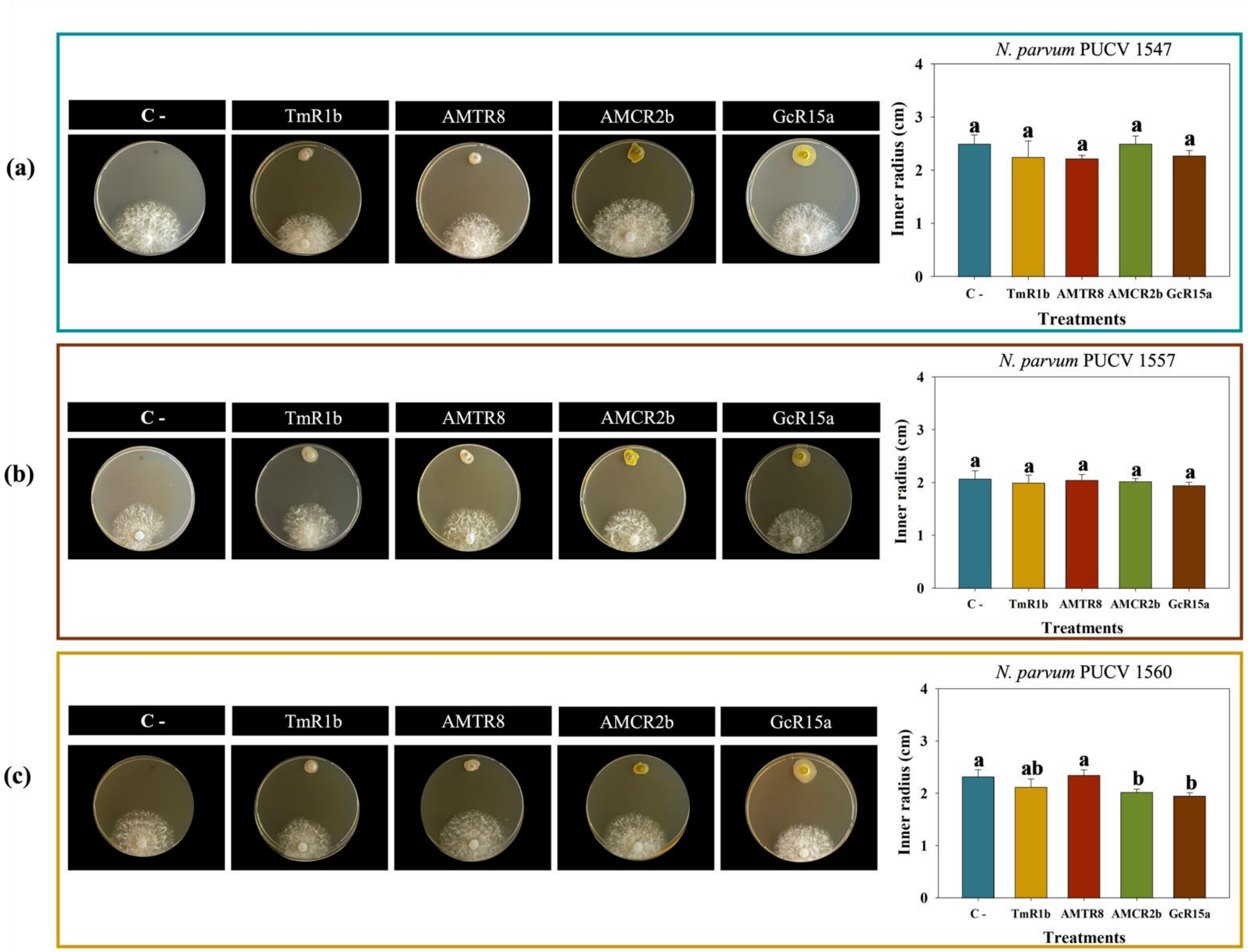

Figure S1. Biocontrol effects of native bacteria on the colony inner radius of *N. parvum* strains after 7 days at 10°C determined using the agar plug diffusion method. Effects on *N. parvum* strains: (**a**) PUCV 1547, (**b**) PUCV 1557, and (**c**) PUCV 1560. Data are presented as mean ± standard deviation (n=4). Statistical significance was determined using Tukey's test ($p \leq 0.05$). Different letters indicate statistically significant differences between treatments. Abbreviations: C-, negative control; TmR1b, *Pseudomonas* sp. TmR1b; AMTR8, *Pseudomonas* sp. AMTR8; AMCR2b, *Pseudomonas* sp. AMCR2b; GcR15a, *Pseudomonas* sp. GcR15a.

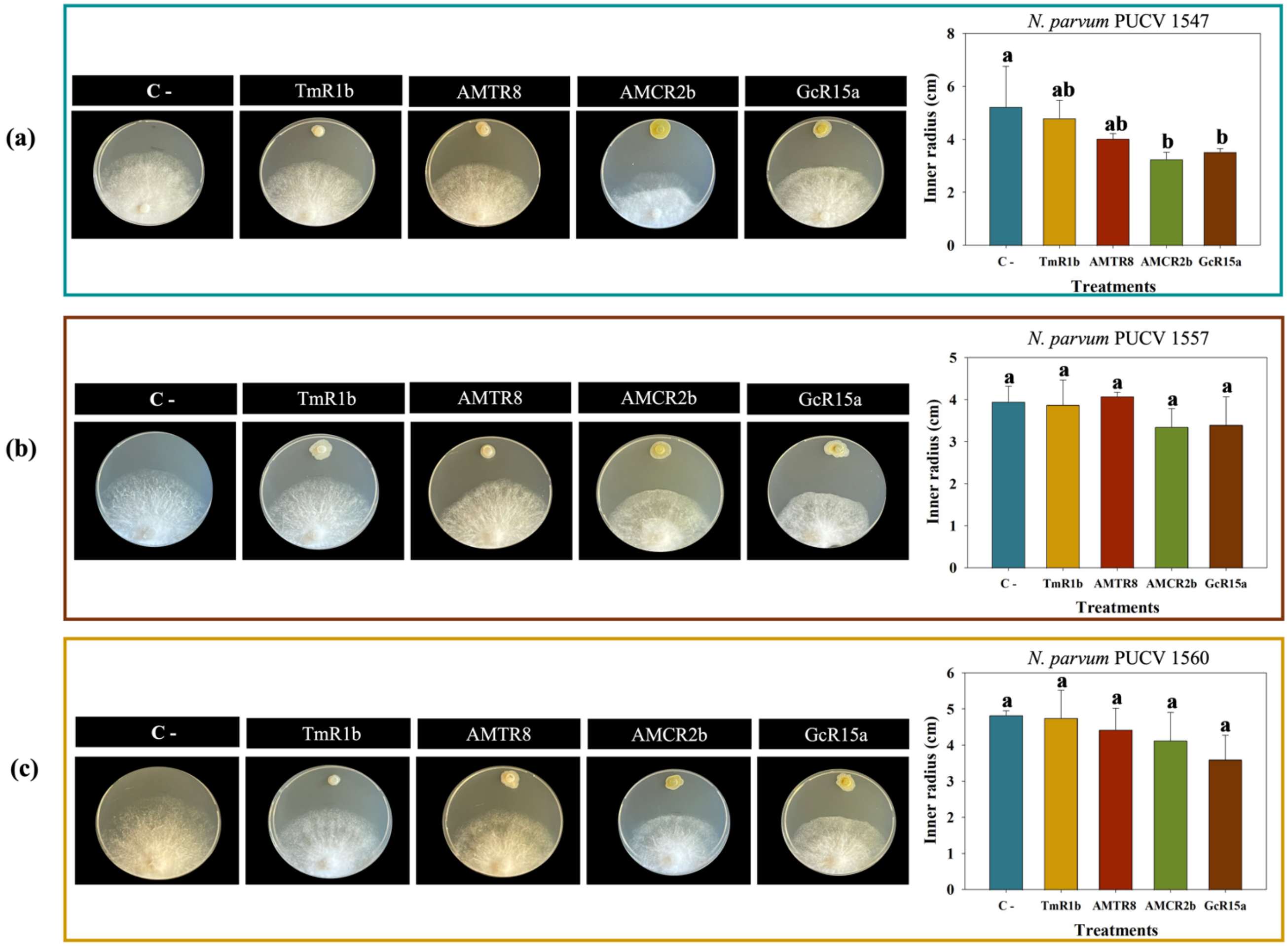

Figure S2. Biocontrol effects of native bacteria on the colony inner radius of *N. parvum* strains after 7 days at 15°C determined using the agar plug diffusion method. Effects on *N. parvum* strains: (**a**) PUCV 1547, (**b**) PUCV 1557, and (**c**) PUCV 1560. Data are presented as mean ± standard deviation (n=4). Statistical significance was determined using Tukey's test ($p \leq 0.05$). Different letters indicate statistically significant differences between treatments. Abbreviations: C-, negative control; TmR1b, *Pseudomonas* sp. TmR1b; AMTR8, *Pseudomonas* sp. AMTR8; AMCR2b, *Pseudomonas* sp. AMCR2b; GcR15a, *Pseudomonas* sp. GcR15a.

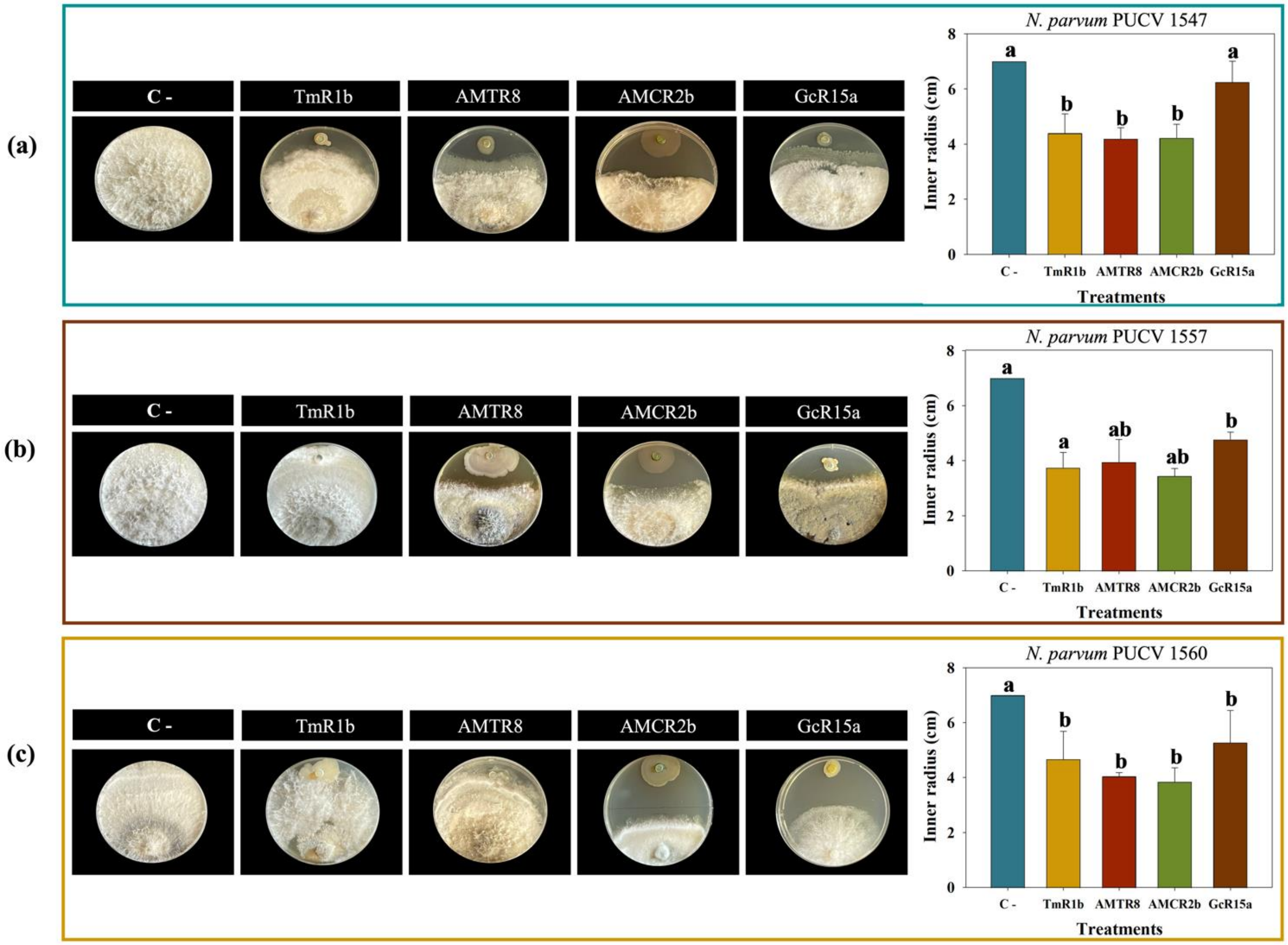

Figure S3. Biocontrol effects of native bacteria on the colony inner radius of *N. parvum* strains after 7 days at 30°C determined using the agar plug diffusion method. Effects on *N. parvum* strains: (**a**) PUCV 1547, (**b**) PUCV 1557, and (**c**) PUCV 1560. Data are presented as mean ± standard deviation (n=4). Statistical significance was determined using Tukey's test ($p \leq 0.05$). Different letters indicate statistically significant differences between treatments. Abbreviations: C-, negative control; TmR1b, *Pseudomonas* sp. TmR1b; AMTR8, *Pseudomonas* sp. AMTR8; AMCR2b, *Pseudomonas* sp. AMCR2b; GcR15a, *Pseudomonas* sp. GcR15a.

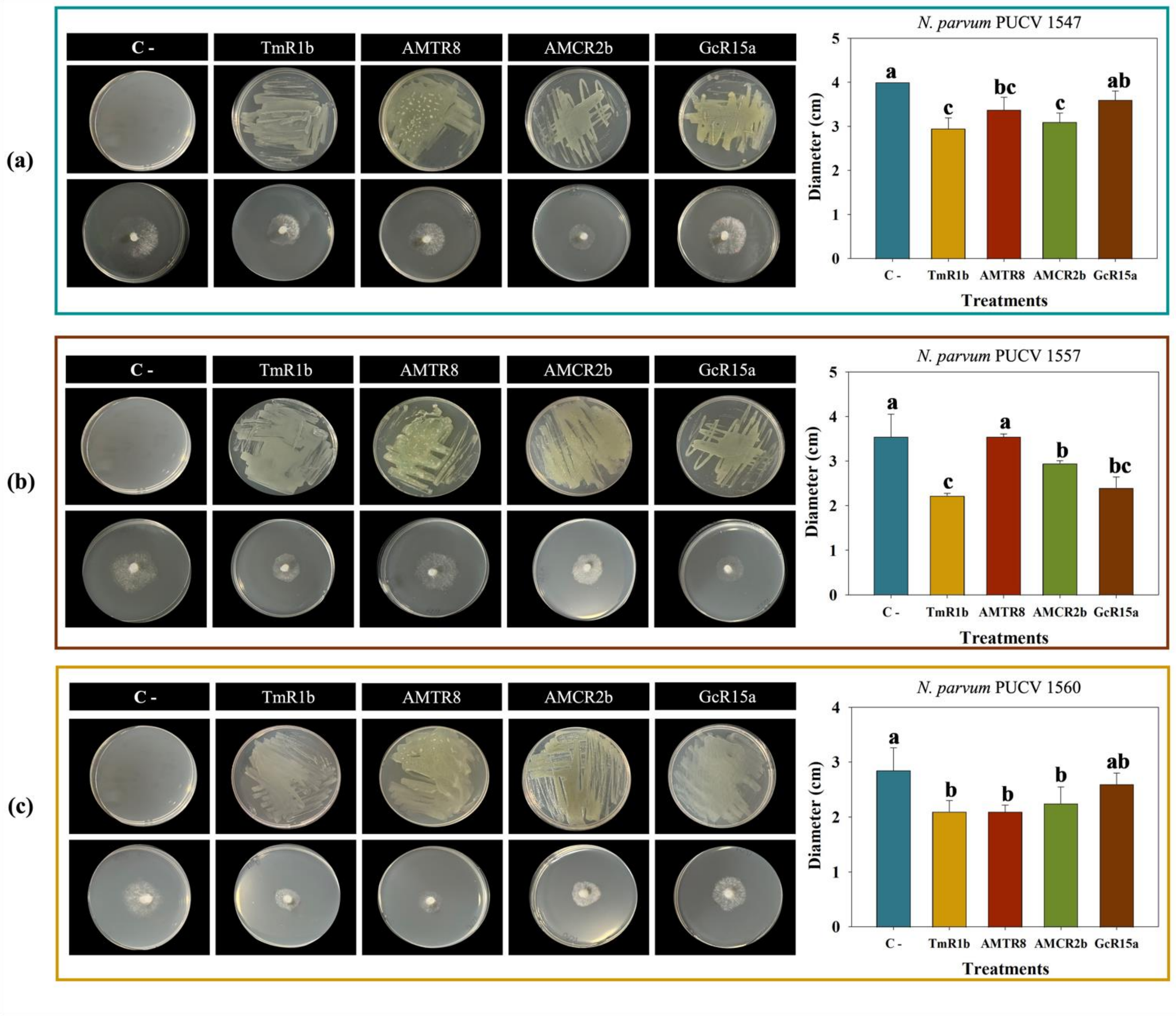

Figure S4. Biocontrol effects of native bacteria on *N. parvum* strains after 72 h at 10°C determined using the double plate method. The fungal colony diameter was measured. Effects on *N. parvum* strains: (**a**) PUCV 1547, (**b**) PUCV 1557, and (**c**) PUCV 1560. Data are presented as mean ± standard deviation (n=4). Statistical significance was determined using Tukey's test ($p \leq 0.05$). Different letters indicate statistically significant differences between treatments. Abbreviations: C-, negative control; TmR1b, *Pseudomonas* spp. TmR1b; AMTR8, *Pseudomonas* sp. AMTR8; AMCR2b, *Pseudomonas* sp. AMCR2b; GcR15a, *Pseudomonas* sp. GcR15a.

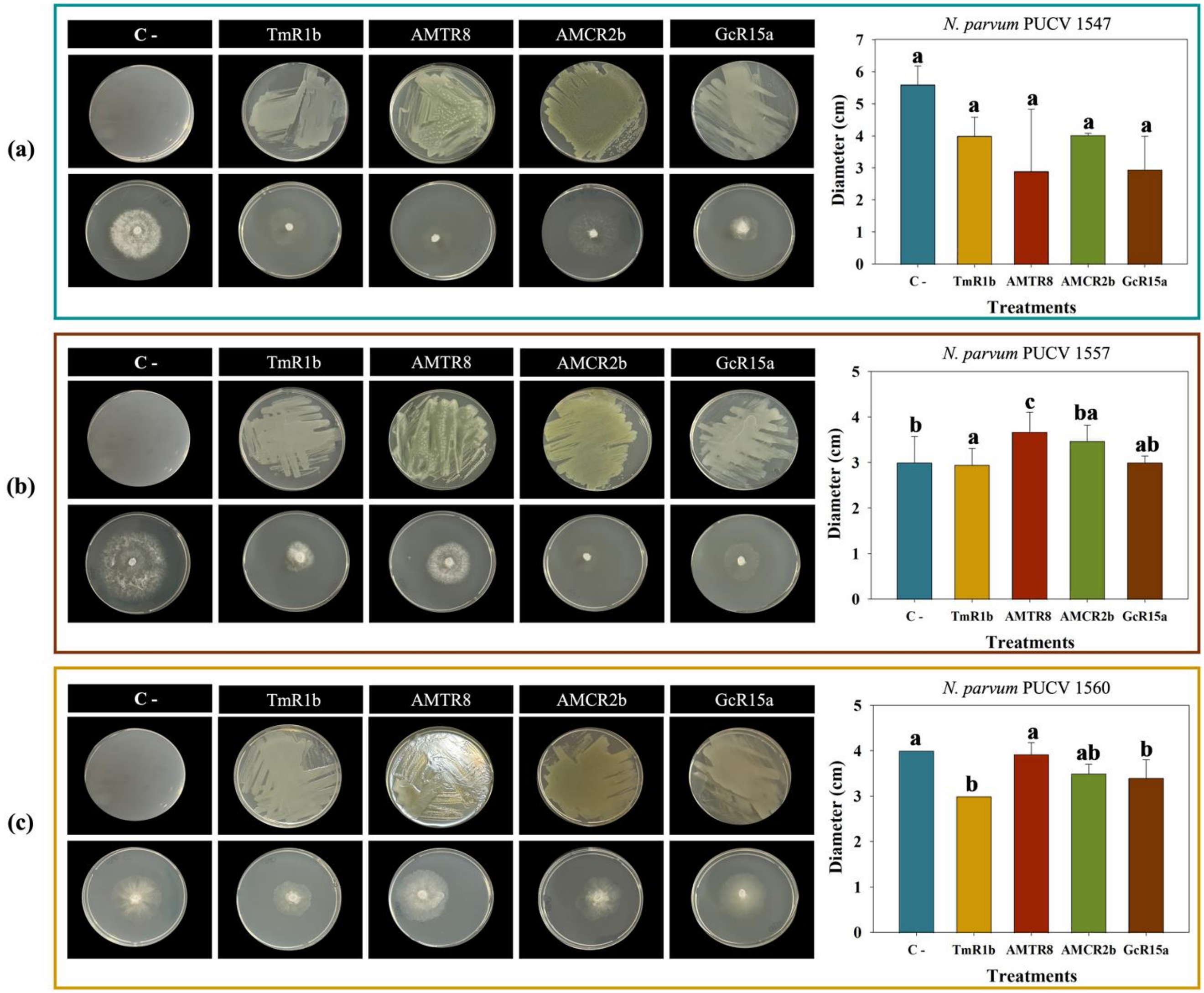

Figure S5. Biocontrol effects of native bacteria on *N. parvum* strains after 72 h at 15°C determined by double plate method. The fungal colony diameter was measured. Effects on *N. parvum* strains: (**a**) PUCV 1547, (**b**) PUCV 1557, and (**c**) PUCV 1560. Data are presented as mean ± standard deviation (n=4). Statistical significance was determined using Tukey's test ($p \leq 0.05$). Different letters indicate statistically significant differences between treatments. Abbreviations: C-, negative control; TmR1b, *Pseudomonas* sp. TmR1b; AMTR8, *Pseudomonas* sp. AMTR8; AMCR2b, *Pseudomonas* sp. AMCR2b; GcR15a, *Pseudomonas* sp. GcR15a.

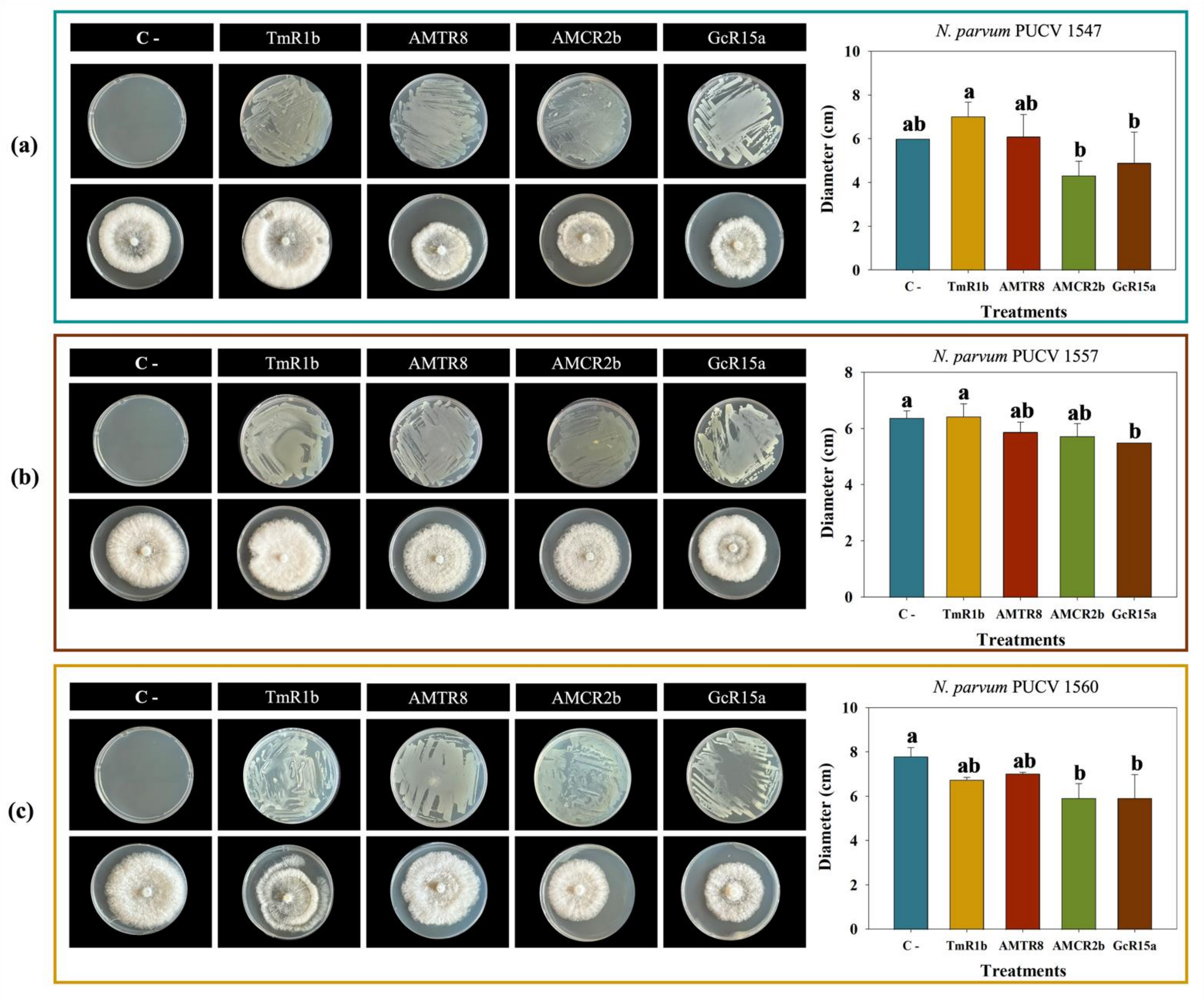

Figure S6. Biocontrol effects of native bacteria on *N. parvum* strains after 72 h at 30°C determined by double plate method. The fungal colony diameter was measured. Effects on *N. parvum* strains: (**a**) PUCV 1547, (**b**) PUCV 1557, and (**c**) PUCV 1560. Data are presented as mean ± standard deviation (n=4). Statistical significance was determined using Tukey's test ($p \leq 0.05$). Different letters indicate statistically significant differences between treatments. Abbreviations: C-, negative control; TmR1b, *Pseudomonas* sp. TmR1b; AMTR8, *Pseudomonas* sp. AMTR8; AMCR2b, *Pseudomonas* sp. AMCR2b; GcR15a, *Pseudomonas* sp. GcR15a.

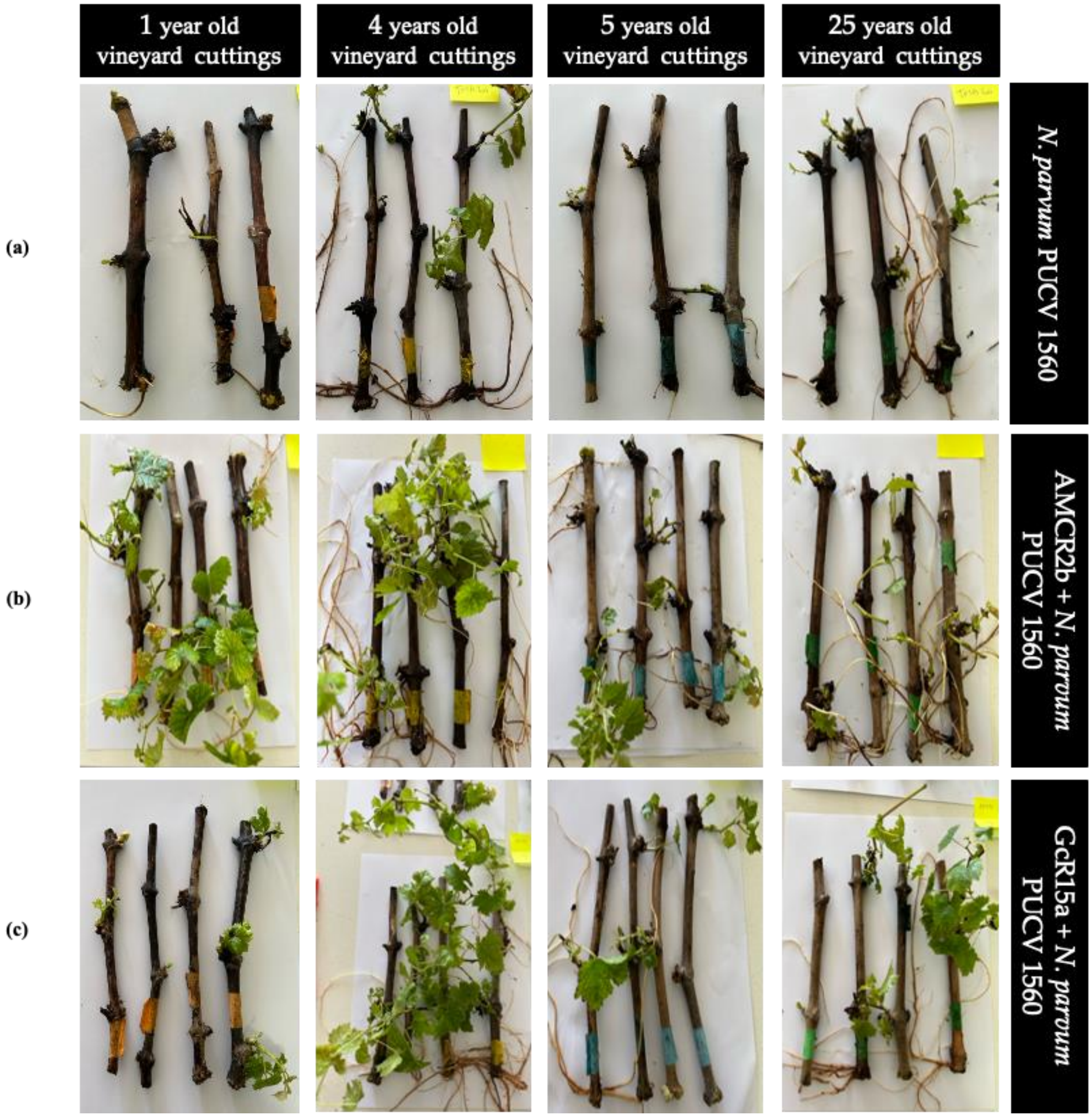

Figure S7. Growth stimulation by *Pseudomonas* sp. strains AMCR2b and GcR15a at 22°C of vineyard cuttings of 1, 4, 5 and 25 years treated with *N. parvum*. Vineyard cuttings of 1, 4, 5 and 25 years are ordered from left to right. (**a**) T0 cuttings were treated with *N. parvum* and not inoculated with bacteria (control). (**b**) T1 cuttings treated with *N. parvum* and inoculated with *Pseudomonas* sp. AMCR2b. (**c**) T2 cuttings treated with *N. parvum* and inoculated with *Pseudomonas* sp. GcR15a.

Table S1. Effects of native bacteria on vascular lesion lengths (cm) caused by *N. parvum* strains in vineyard cuttings of different ages.

Pathogen	Vineyard age (years)	C (+)	Strain AMCR2b	Strain GcR15a	C (-)
N. parvum PUCV 1547	1	0 ± 0.0 [a]	0.87 ± 1.44 [a]	0.22 ± 1.85 [a]	3.64 ± 3.64 [a]
	4	0 ± 0.0 [a]	0.50 ± 1.00 [a]	0.87 ± 0.75 [a]	1.87 ± 1.93 [a]
	5	0 ± 0.0 [a]	0.37 ± 0.48 [a]	1.13 ± 1.65 [a]	1.25 ± 1.26 [a]
	25	0 ± 0.0 [b]	0.20 ± 0.24 [b]	0.10 ± 0.14 [b]	1.00 ± 0.41 [a]
N. parvum PUCV 1557	1	0 ± 0.0 [a]	0.73 ± 0.34 [a]	0.72 ± 0.87 [a]	1.03 ± 1.66 [a]
	4	0 ± 0.0 [a]	0.3 ± 0.34 [a]	0.20 ± 0.24 [a]	0.68 ± 0.91 [a]
	5	0 ± 0.0 [b]	0.15 ± 0.13 [ab]	0.15 ± 0.24 [ab]	1.56 ± 1.39 [a]
	25	0 ± 0.0 [a]	0.25 ± 0.5 [a]	0.08 ± 0.15 [a]	0.48 ± 0.39 [a]
N. parvum PUCV 1560	1	0 ± 0.0 [a]	0.24 ± 0.21 [a]	2.13 ± 1.65 [a]	5.15 ± 8.57 [a]
	4	0 ± 0.0 [b]	0.25 ± 0.5 [b]	0.46 ± 0.07 [b]	1.75 ± 0.96 [a]
	5	0 ± 0.0 [a]	0.10 ± 0.14 [a]	0.45 ± 0.42 [a]	0.60 ± 0.49 [a]
	25	0 ± 0.0 [a]	0.23 ± 0.22 [a]	0.45 ± 0.42 [a]	1.13 ± 1.44 [a]

Data are expressed as mean ± standard deviation (n=6).

Letters indicate statistically significant differences compared to the control (Tukey test, $p \leq 0.05$).

Table S2. Effects of native bacteria on vascular lesion lengths (cm) caused by *N. parvum* strains in 7-year-old vineyard shoots.

Variety	*N. parvum* strain	Vineyard age (years)	C (+)	Bacterial Consortium	Strain PU4	C (-)
Cabernet Sauvignon	PUCV 1547	7	0.63 ± 0.1 [c]	1.25 ± 0.2 [c]	2.15 ± 0.2 [b]	6.1 ± 0.8 [a]
	PUCV 1557		0.72 ± 0.2 [c]	1.08 ± 0.2 [c]	3.1 ± 0.3 [b]	3.9 ± 0.3 [a]
	PUCV 1560		0.6 ± 0.4 [b]	1.1 ± 0.6 [b]	1.73 ± 0.3 [b]	8.2 ± 8.57 [a]
Sauvignon Blanc	PUCV 1547	7	0.63 ± 0.1 [b]	1.25 ± 0.1 [b]	2.15 ± 0.6 [b]	12.5 ± 2. [a]
	PUCV 1557		0.15 ± 0.2 [c]	3.98 ± 0.3 [b]	4.08 ± 0.4 [b]	5.5 ± 0.2 [a]
	PUCV 1560		3.88 ± 0.3 [a]	1.13 ± 0.5 [c]	2.88 ± 0.6 [b]	4.3 ± 8.57 [a]

Data are expressed as mean ± standard deviation (n=5).

Letters indicate statistically significant differences compared to the control (Tukey test, $p \leq 0.05$).
